# Upward trends of acquired drug resistances in Ethiopian HIV-1C isolates: A decade longitudinal study

**DOI:** 10.1371/journal.pone.0186619

**Published:** 2017-10-19

**Authors:** Andargachew Mulu, Melanie Maier, Uwe Gerd Liebert

**Affiliations:** 1 Armauer Hanssen Research Institute (AHRI), Addis Ababa, Ethiopia; 2 Institute of Virology, Medical Faculty, Leipzig University, Leipzig, Germany; National and Kapodistrian University of Athens, GREECE

## Abstract

**Background:**

The emergence, accumulation and spread of HIV-1 drug resistance strains in Africa could compromise the effectiveness of HIV treatment programs. This study was aimed at determining the incidence of virological failure and acquired drug resistance mutations overtime and identifying the most common mutational pathways of resistance in a well characterized HIV-1C infected Ethiopian cohort.

**Methods:**

A total of 320 patients (220 ART naïve and 100 on first lines ART) were included and followed. ART initiation and patients’ monitoring was based on the WHO clinical and immunological parameters. HIV viral load measurement and genotypic drug resistance testing were done at baseline (T0-2008) and after on average at a median time of 30 months on ART at three time points (T1-2011, T2-2013, T3-2015).

**Findings:**

The incidence of virological failure has increased overtime from 11 at T1 to 17 at T2 and then to 30% at T3. At all time point’s almost all of the patients with virological failure and accumulated drug resistance mutations had not met the WHO clinical and immunologic failure criteria and continued the failing regimen. A steep increase in the incidence and accumulation of major acquired NRTI and NNRTI drug resistance mutations have been observed (from 40% at T1 to 64% at T2 and then to 66% at T3). The most frequent NRTIs drug resistance associated mutations are mainly the lamivudine-induced mutation M184V which was detected in 4 patients at T1 and showed a 2 fold increase in the following time points (T2: n = 8) and at (T3: n = 12) and the thymidine analogue mutations (such as D67N, K70R and K219E) which were not-detected at baseline T0 and T1 but were increased progressively to 10 at T2 and to 17 at T3. The most frequent NNRTIs associated mutations were K103N, V106M and Y188C.

**Conclusions:**

An upward trend in the incidence of virological failure and accumulation of NRTI and NNRTI associated acquired antiretroviral drug resistance mutations are observed. The data suggest the need for virological monitoring, resistance testing for early detection of failure and access for TDF and PI containing drugs. Population-level and patient targeted interventions to prevent the spread of mutant variants is warranted.

## Introduction

In Africa, over the past decade a remarkable increase in access to combination antiretroviral therapy (cART) has been continued and has substantially contributed to the dramatic reduction in morbidity and mortality. It has also improved quality of life and life expectancy and altered perceptions on HIV/AIDS from an epidemic to a manageable chronic illness [1, 2]. These achievements are based on the WHO public health approach of using clinical and immunological parameters as the standard for ART initiation and monitoring, despite its limitations compared with the reference virological parameters based approach [3, 4]. The exclusive use of this simplified strategy to initiate and switch ART together with the use of drugs with low-genetic-barrier and the socio-cultural issues in the region may favor the rapid emergence, accumulation and transmission of resistant viruses.

Along with the expanded coverage of ART, prevalence of transmitted drug resistance (TDR) is rising overtime [5–7] predominantly associated with nonnucleoside reverse transcriptase inhibitors (NNRTIs). Moreover, few available studies on long term virological outcomes showed that virological failure remains undetected and patients continue to be on failing regimen until either clinical or immunological failure occurs [8] which leads to high rates of treatment failure with virological failure rates of greater than 20% after 24 months of ART and development of resistance with the potential of reducing the efficacy of available first-line regimens [8, 9] in up to 90% of patients and being considered as a major threat for ongoing and future treatment options[8–12]. Although many studies have reported early outcomes of patients on ART, they were limited to patient’s outcomes in terms of retention and survival [13] without baseline virological data.

In Ethiopia, according to the recent report from the Federal HIV Prevention and Control Office, there are about 740,000 people living with HIV in Ethiopia, and only 400,000 are receiving antiretroviral treatment. Previous studies in early years of HIV and recent studies including our previous works show the presences of HIV-1C clade homogeneity in 99% of Ethiopian isolates [14]. ART in Ethiopia consists of generic low cost fixed-dose combination (FDC) of two NRTI and

one NNRTI with first line regimens of lamivudine (3TC) combined with stavudine (d4T) or zidovudine (AZT), and either nevirapine (NVP) or efavirenz (EFV) permitting four alternative treatment regimens: D4T+3TC+NVP; D4T+3TC+EFV; ZDV+3TC+NVP; or ZDV+3TC+EFV. For second line therapy, the nucleoside backbone is changed to ABC, TDF, ddI or ZDV (if not used in first-line therapy); and, in addition, the non-nucleoside reverse transcriptase inhibitors (NNRTIs) NVP or EFV was replaced by one of the boosted protease inhibitors (PIs) lopinavir (LPV/r), saquinavir (SQV/r) or indinavir (IND/r) [https://aidsfree.usaid.gov/]. Although D4T has been omitted from ART recommendations because of its side effects in 2008, it is still used extensively in Ethiopia. ART initiation and monitoring is based on the WHO clinico-immunological approach. However, as this approach lacks viral load determination and drug resistance testing HIV infected individuals may be at risk for “unrecognized” virologic failure and the subsequent development of antiretroviral drug resistance.

In this a decade longitudinal study, we aimed to determine the incidence of virological failure and acquired drug resistance mutations overtime and to identify the most common mutational pathways of resistance among two groups of patients who had initiated ART at the scale-up (2008)-Group 1 and first roll out (2005)—Group 2 of ART in a well characterized HIV-1C infected Ethiopian cohort [3, 4, 14].

## Methods

### Study design

**Fig 1:**
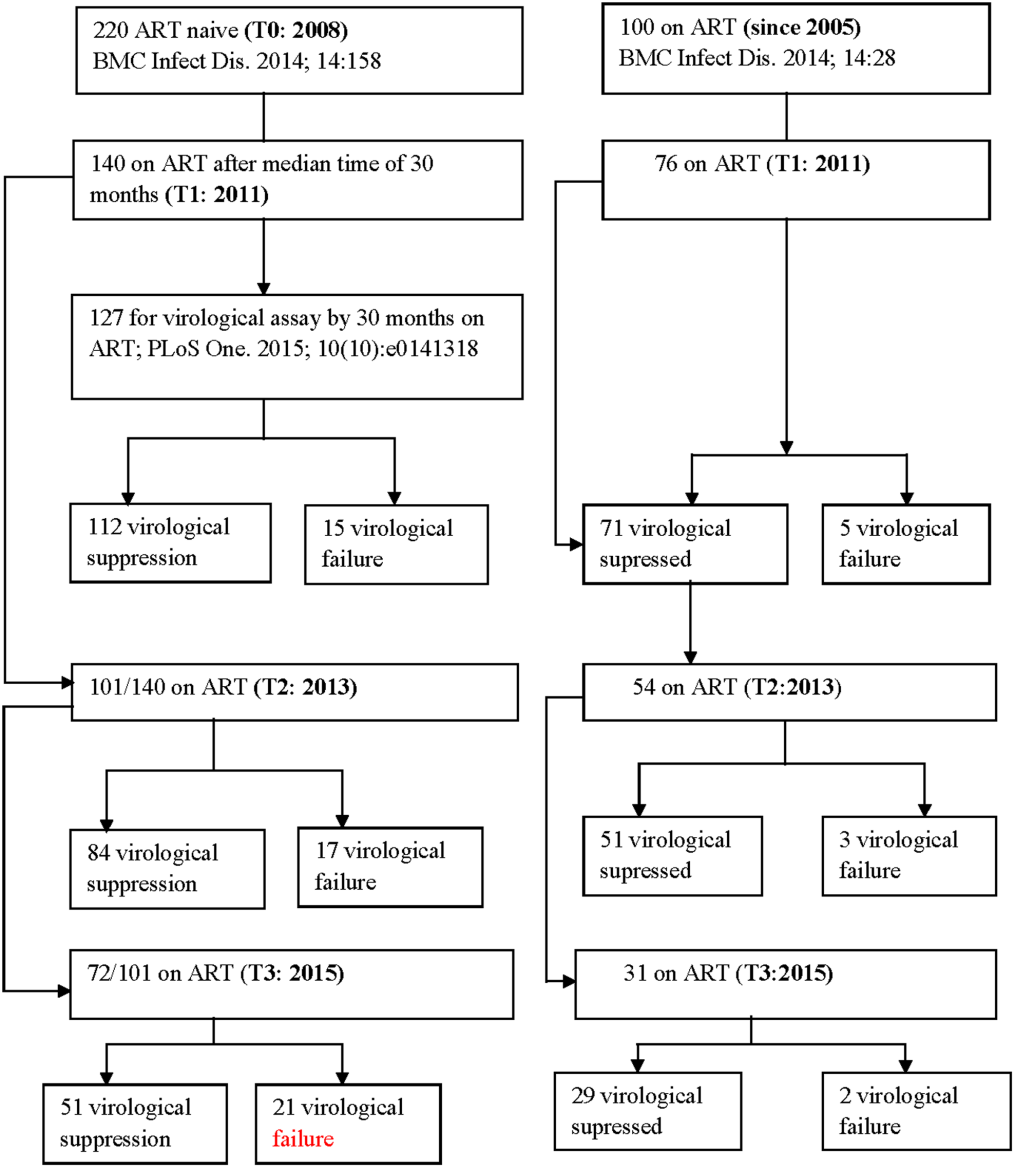
Cohort profile.

This is a prospective cohort study where the baseline characteristics [3, 14] of the groups and the outcome of ART after a median time of 30 months [4] have been described before. The cohort profile is depicted in Fig 1 The study protocol and design including the consent procedures were approved by the University of Gondar Ethical Review Committee (RPO/55/291/00).

### Treatment and patients monitoring

As described before [3, 4, 14], patients were evaluated with a standardized form at enrolment and diagnosed based on the WHO criteria of AIDS-defining conditions. Although, the eligibility criterion for ART has been changed over the years the initial criteria based on CD4 count <200 cells/mm^3^or WHO stage 4 was used [13].

### Blood collection and laboratory investigations

Sample collection and preparation, CD4^+^ T cells count, HIV-1 RNA extraction, pol gene amplification and sequencing were done as described previously [3, 4, 14]. Briefly, blood samples was collected in vacutainer tubes containing ethylene diamine tetraacetic acid (EDTA) at baseline, every 6 months (for CD4^+^ T cells count only) and at last by a median time of 24 months on ART for viral load determination and detection of drug resistance mutations. Immunological failure was defined as failure to achieve a CD4^+^ T cells gain of at least 50 cells above pre-therapy level or having an absolute CD4^+^ T cells count of < 100 cells/mm^3^ after one year of therapy [15].

A baseline blood samples refers to a blood sample collected prior to the initiation of ART and denoted by T0 (2008). The time point T1 was a blood sample collected in a median time of 30 months on ART (2011) and the time points T2 (2013) and T3 (2015) were blood samples collected at least in a median time of 24 months. Thus, HIV viral load was measured at T0 (N = 220), T1 (N = 127), T2 (N = 101) and T3 (N = 72) for Group-2 and at T1 (N = 100), T2 (N = 67) and T3 (N = 44) for Group-1 patients. Although, WHO defines virological failure using viral load cut-offs of > 3log copies/ml after 3 months with adherence support [15], there is no standardized reporting of virologic failure since 3 months may not enough for full viral suppression and since resistance mutations could be detected in samples with VL of as low as 2.5log copies/ml and not in patients with viral load of >1000 copies/ml. Thus, in this study viral load of below the detection limits of the assay (1.6log copies/ml), between 1.6 and 2.6log copies/ml and >2.6log copies/ml in single plasma sample were defined as virologic suppression, low-level viremia and virological failure, respectively [16]. Genotypic drug resistance mutations were interpreted according to the Stanford University drug resistance database (http://hivdb.stanford.edu) and the 2017 IAS mutation list [17]. The Stanford HIV Drug Resistance Database (HIVDB) mutation penalty scoring system (http://hivdb.stanford.edu/DR/) was also used to estimate genotypic susceptibility scores (GSSs) and characterize the level of resistance in clinical isolates. HIV-1 subtype was determined by using the REGA HIV-1 Automated Subtyping Tool version 2 (http://www.bioafrica.net).

## Results

### Cohort characteristics of Group 1

The level of transmitted drug resistance at baseline (T0) was found to be 5.6% and 13.1% according to the Stanford University HIVDB drug resistance interpretation algorithms and the International Antiviral Society mutation lists, respectively (S1 Table). Furthermore, viral suppression rate after a median time of 30 months on ART (T1) was found to be 88.2% (112/127). Among the 15 virologically failed patients, six harbored one or more drug resistant associated mutations in the reverse transcriptase region (S2 Table).

### Incidence of virological failure

**Fig 2:**
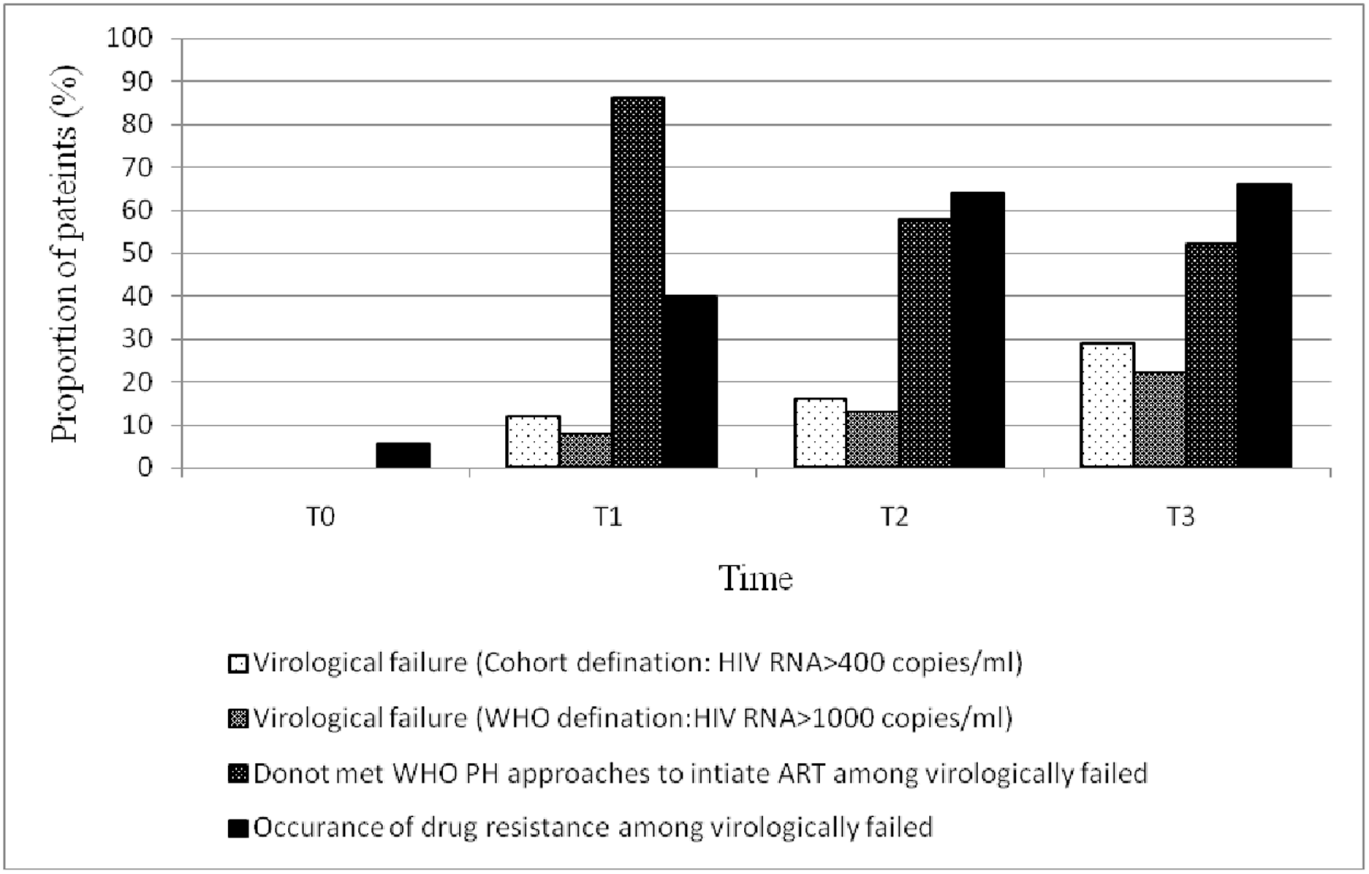
Incidence of virological failure and acquired drug resistance over time.

In this study, by the time point T2 (after 5 years on ART) and T3 (after 8 years on ART), 72% (101/140) and 71% (72/101) were found to be active on ART program. At T2, among those on ART a total of 83% (84/101) achieved virological suppression which with time decreased to 70% (51/72) by the time point T3. In another words, the incidence of virological failure has increased overtime from 11% at T1 [4] to 17% at T2 and then to 30% at T3 (Fig 2). Moreover, an increasing median HIV-1 RNA level in virologically failed subjects was observed over time (4.28, 4.42 and 5.4log10 copies/ml at T1, T2 and T3, respectively. Elven out of 15 (73%), 11/17 (64%) and 15/21(71%) of the patients with cohort definition of VF (above 1.6log10 copies/ml) met the WHO virological failure criteria (>3log10 copies/ml) at time point T1, T2 and T3, respectively. However, at all time points almost all of the patients with virological failure and accumulated drug resistance mutations had not met the WHO clinical and immunologic failure criteria and continued the failing regimen although a reduction in CD^+^4 T cell counts is noted (Tables 1 and 2). At time point T1 drug resistance mutations were not detected in 2 patients despite high viral load (S2 Table). This phenomenon was seen in 6 patients each at time point T2 (Table 1) and T3 (Table 2). About a quarter of the patients who did not reach virological suppression at T2 and T3 had had a low-level viremia at the preceding time points.

**Table 1:** Clinical characteristics and antiretroviral drug resistance mutation (at time point-T2).

ID	Age*/Sex	CD4^+^ cells			HIV-1 RNA			ART Regimen^1^			Time	NRTIs		NNRTIs	
		T0	T1	T2	T0	T1	T2	T0	T1	T2		Mutations	Resistant^2^	mutations	Resistant^2^
0003	48/M	251	291	221	5.91	ud	6.04	1a	1a	1a	68	M184V, V75I, K65R, Y115F	3TC, FTC, ABC, TDF, D4T	K103N,L100I, V179T	EFV, NVP, RPV, *ETR*
0005	38/M	201	249	211	3.85	ud	5.63	1a	1a	1c	63	M184V, T215Y	3TC, FTC, ABC, TDF, *AZT*, *D4T*	V106V, F227L	EFV, NVP
0006	43/F	81	378	289	5.04	ud	5.50	1a	1c	1a	62	None	-	Y181L, P225H	EFV, NVP, RPV
0009	48/F	178	347	293	4.50	ud	5.69	1a	1c	1a	60	K65R	TDF, DDI, *ABC*, *D4T*	K103N, V106M	EFV, NVP
0013	38/F	184	299	168	4.05	ud	3.92	1a	1a	1c	67	K65R, Y115F, M184V	3TC, FTC, ABC, TDF, D4T, FTC, *D4T*	K101E, Y181C, G190A	EFV, NVP, RPV, ETR
0014	38/F	76	399	383	4.31	2.65	4.63	1a	1a	1a	66	M184V	3TC, FTC	K103N, E138K, P225H, K238T	EFV, NVP, ETR, *RPV*
0017	44/F	84	357	121	5.79	ud	5.37	1a	1b	1d	69	E44A, L74I, K70R, M184V, V118I, T215V, K219E, L210W	3TC, ABC, AZT, D4T, DDI, FTC, *TDF*	K103N	EFV, NVP
0018	35/F	149	358	277	5.01	2.57	5.79	1a	1a	1d	59	M184I, L74I, D67N	3TC, ABC, D4T, FTC	G190E	EFV, NVP, RPV, ETR
0019	49/F	293	421	302	5.43	ud	6.04	1a	1a	1c	65	M184I, L74I, D67N	3TC, ABC, D4T, FTC	V90I, G190E	EFV, NVP, RPV, ETR
0039	47/M	181	396	417	4.42	ud	4,46	1a	1c	1c	64	T69D	DDI	None	-
0035	39/M	219	467	311	3.03	2.19	5.30	1a	1c	1c	62	D67N, T69D, K70R, M184V, K219Q	3TC, DDI, FTC, *ABC*, *AZT*, *D4T*	V106M, Y188C	EFV, NVP
0001	39/M	189	356	341	5.34	ud	3.74	1a	1a	1c	58	None	-	None	-
0004	43/M	162	325	299	5.56	ud	4,66	1a	1a	1c	61	None	-	None	-
0007	41/M	195	399	311	4.98	2.39	3.93	1b	1b	1c	57	None	-	None	-
0011	37/M	230	379	302	6.12	ud	2.19	1a	1c	1c	67	None	-	None	-
0020	45/F	211	496	349	5.29	ud	5.60	1b	1c	1c	67	None	-	None	-
0022	29/F	165	389	377	5.47	ud	5.19	1b	1c	1c	59	None	-	None	-

* Age (years) is at time point (T2);

CD4 T cells/mm3;

HIV-1 RNA log10 copies/ml;

Time on ART (months);

ud: HIV RNA< 1.6log10 copes/ml;

^1^1a: 3TC—d4T + NVP; 1b: 3TC—d4T + EFV; 1c: 3TC—AZT+ NVP; 1d: 3TC—AZT + EFV;

^2^3TC (Lamivudine), d4T (Stavidine), NVP (Nevirapine), EFV (Efavirenz), AZT (Zidovidine); Intermediate Level of Resistance to the drugs in *italic* type.

**Table 2:** Clinical characteristics and antiretroviral drug resistance mutation (at time point- T3).

ID	Age*/Sex	CD4^+^ T				HIV RNA				ART^1^				Time	NRTI		NNRTI	
		T0	T1	T2	T3	T0	T1	T2	T3	T0	T1	T2	T3		Mutations	Resistant^2^	mutations	Resistant^2^
0023	51/M	210	342	388	291	4.43	ud	ud	5.59	1a	1a	1a	1a	65	M184V, M41L, T215I	3TC, FTC, *ABC*, *AZT*, *D4T*, *DDI*	K101Q, V106A, F227L	EFV, NVP
0026	28/M	201	287	345	149	4.17	ud	ud	3.46	1a	1a	1a	1a	67	M184I	3TC, FTC	V106M, G190A, V179D, F227L, V90I	EFV, NVP, ETR, *RPV*
0027	25/F	229	367	411	293	5.43	ud	2.58	5.10	1c	1c	1c	1c	56	M184V, D67N, K70R, T69D, K219Q	3TC, DDI, FTC, *ABC*, *AZT*, *D4T*	V106M, Y188C	EFV, NVP
0028	29M	199	325	412	181	4.42	ud	ud	5.16	1c	1c	1c	1c	59	D67N, T69D, K70R, M184V, K219Q	3TC, DDI, FTC, *ABC*, *AZT*, *D4T*	V106M, Y188C	EFV, NVP
0030	38/F	274	378	411	219	3.03	ud	2.59	5.36	1c	1c	1c	1c	66	M184V, K70R	3TC, FTC, *ABC*	G190A, K101E, Y181C, H221Y	EFV, NVP, ETR, RPV
0032	30/M	32	149	301	199	3.76	ud	ud	4.37	1c	1c	1c	1c	54	K65R, Y115F, M184V	3TC, ABC, DDI, FTC, TDF, *D4T*	Y181C	NVP, *EFV*, *ETR*, *RPV*
0033	28/M	191	289	389	357	4.43	ud	ud	4.96	1c	1c	1c	1c	67	K70E, L74I, Y115F, M184V	3TC, ABC, DDI, FTC, *TDF*	V106M, V179D, H221Y, F227L	EFV, NVP
0034	25/F	149	276	432	458	4.17	ud	2.42	6.47	1d	1d	1d	1d	71	D67N, K70R, M184V, T69Q, K219Q	3TC, DDI, FTC, *ABC*, *D4T*, *AZT*	V106M, Y188C	EFV, NVP
0037	38/F	181	265	378	396	4.42	ud	2.54	6.20	1d	1d	1d	1d	63	D67N, T69D, K70R, M184V, K219Q	3TC, DDI, FTC, *ABC*, *D4T*, *AZT*	V106M, Y188C	EFV, NVP
0040	30/M	219	329	399	467	3.03	ud	ud	2.94	1a	1a	1a	1a	59	K70R, M184V	3TC, FTC	V106M, Y188C	EFV, NVP
0041	51/M	258	287	381	291	4.43	ud	ud	3.04	1b	1b	1b	1b	57	D67N, T69D, K70R, M184V, K219Q	3TC, FCT, DDI, *ABC*, *D4T*, *AZT*	V106M, Y188C	EFV, NVP
0046	28/F	198	342	358	349	4.17	ud	ud	2.67	1c	1c	1c	1c	72	M184V	3TC, FTC	K103N	EFV, NVP
0052	25/F	149	276	276	293	5.43	ud	ud	3.99	1c	1c	1c	1c	69	None	-	K103N	EFV, NVP
0012	25/F	62	345	341	270	2.20	ud	ud	3.00	1a	1a	1a	1a	61	None	-	K103N	EFV, NVP
0008	29/F	193	257	349	421	5.43	ud	ud	2.94	1a	1a	1a	1a	66	None	-	K103N	EFV, NVP
0024	37/F	176	267	421	476	5.44	ud	2.50	5.59	1b	1b	1b	1b	69	None	-	None	-
0025	41/M	169	325	388	347	4.78	ud	ud	3.00	1c	1c	1c	1c	70	None	-	None	-
0029	38/F	201	298	349	399	4.99	ud	2.25	5.45	1c	1c	1c	1c	57	None	-	None	-
0031	35/M	69	178	289	364	6.20	ud	ud	4.23	1c	1c	1c	1c	62	None	-	None	-
0038	45/F	196	287	388	345	4.77	ud	ud	3.14	1d	1d	1d	1d	68	None	-	None	-
0054	39/M	184	321	428	369	5.22	ud	ud	4.28	1c	1c	1c	1c	62	None	-	None	-

* Age (years) is at time point (T2);

CD4 T cells/mm3;

HIV-1 RNA log10 copies/ml;

Time on ART (months);

ud: HIV RNA< 1.6log10 copes/ml;

^1^1a: 3TC—d4T + NVP; 1b: 3TC—d4T + EFV; 1c: 3TC—AZT + NVP; 1d: 3TC—AZT + EFV;

^2^3TC (Lamivudine), d4T (Stavidine), NVP (Nevirapine), EFV (Efavirenz), AZT (Zidovidine); Intermediate Level of Resistance to the drugs in *italic* type.

### Incidence of acquired drug resistance mutation

None of the patients who had acquired drug resistance associated mutations had presented pre ART drug resistance mutations suggesting that these mutations are developed after treatment initiation and are definitely acquired with obvious limitation of population based sequencing which miss minority resistant variants. There was high sequence similarity and strong clustering between plasma samples of individual patients at different time points (Data not shown). Among individuals with virological failure a steep increase in the incidence of accumulation of major acquired NRTI and NNRTI drug resistance mutations have been observed overtime (from 40% (6/15) at T1 to 64% (11/17) at T2 and then to 66% (15/21) at T3 (Fig 2).

**Fig 3:**
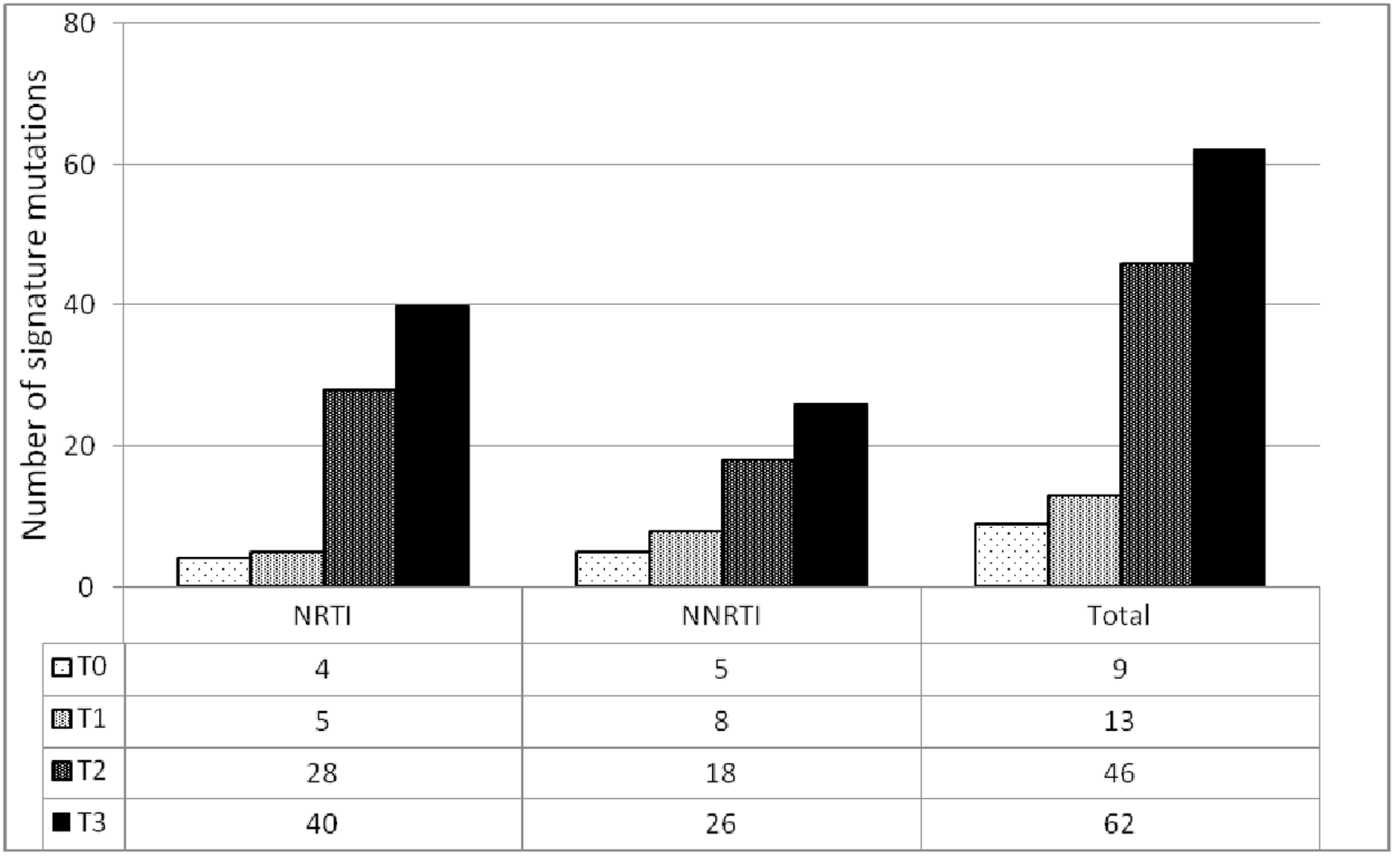
Accumulation of acquired drug resistance mutations over time.

At baseline (T0), 4 NRTIs transmitted drug resistance associated mutations were observed. By the time point T1, 5 NRTIs associated mutations were observed which then increased substantially to 28 by the time point T2 and further increased to 40 by the time point T3 (Fig 3).

**Fig 4:**
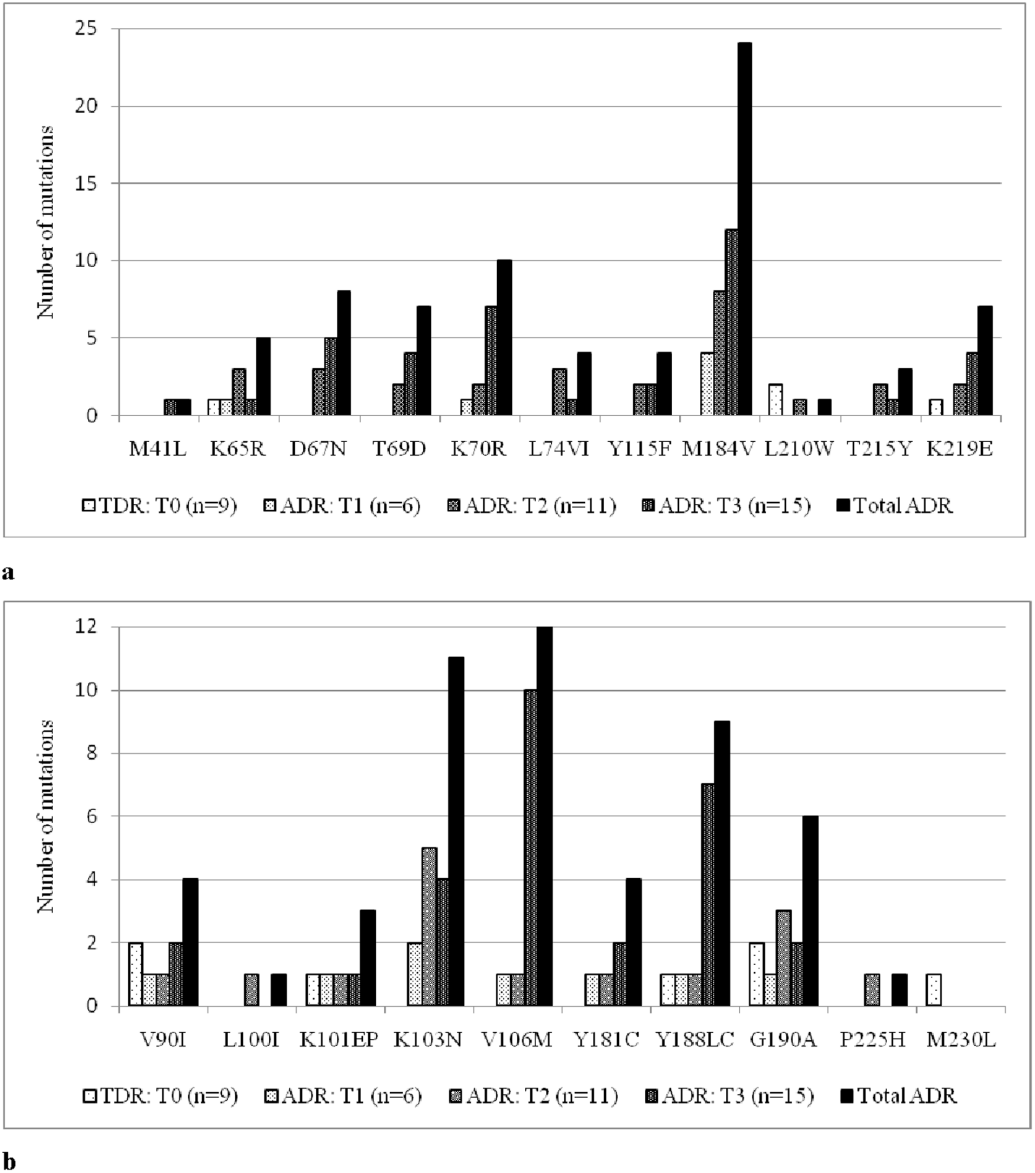
Accumulation of NRTI (a) & NNRTIs (b) associated acquired drug resistance mutations over time.

The most frequent NRTIs drug resistance associated mutation is mainly lamivudine-induced mutation M184V which was detected in 4 patients at T1 and showed a 2 fold increase in the following time points (T2: n = 8) and at (T3: n = 12). The occurrence of thymidine analogue mutations (TAMs) were not-detected at baseline T0 and T1 but was increased progressively to 10 at T2 and to 17 at T3. Among the two TAMs pathways, TAM-2 which features D67N, K70R, T215F, and K219E/Q mutations was more common than the TAM-1 which features M41L, L210W and T215Y. Most of the patients with multiple TAMs were on d4T or AZT containing regimen (Tables 1 and 2). At all-time points, relatively low frequency of K65R mutation was found (Fig 3). Mutations conferring multi-drug resistance such as Q151M and T69ins were not detected. However, additional mutational pathway at codon 69 from T to D known to reduce susceptibility to D4T and DDI has been observed at time point T2 (n = 3) and T3 (n = 4) (Fig 4a).

At baseline (T0), 5 NNRTIs transmitted drug resistance associated mutations were observed. At T1, the NNRTIs resistance associated mutations were detected at a lower frequency [K103N (n = 2), V106M, Y181S, Y188L, V90I, K101E and G190A (n = 1 each)]. But with time like NRTIs, the frequency and pattern of NNRTIs associated mutations increased significantly. Briefly, by the time point T1, 8 NNRTIs associated mutations were observed which then increased substantially to 18 by the time point T2 and further increased to 26 by the time point T3 (Fig 3). The most frequent

Dual-class resistance to NRTIs and NNRTIs were not detected at baseline T0 (S1 Table). But eventually observed in 5/8, 8/11 and 12/15 patients at time point T1 (S2 Table), T2 (Table 1) and T3 (Table 2), respectively with frequent combination of M184V and NNRTI resistance associated mutations. Most of the viral isolates had developed resistance to drugs that the patients were receiving frequently for NRTIs like lamuvidine/emtricitabine, zidovidine, stavudine or tenofovir and for NNRTIs like nevirapine or efavirenz. But still few isolates had reduced susceptibility or resistant to other drugs that the patients did not receive (Tables 1 and 2).

Major drug resistance mutations on PR region were not detected at all time points. However, in the majority of the patients at all time points, several secondary mutations that may facilitate the development of PI resistance were found at higher frequency which could be naturally occurring minor mutations/polymorphic changes (positions M36I, R41K, H69K, L89M, and I93L) but their clinical significance is uncertain. Seven out of eleven at T1 and 9/15 at T2 who had treatment switch to second line ART (PI/r + 2NRTIs regimen) were found to be virologically suppressed.

### Genotypic susceptibility score (GSS)

**Fig 5:**
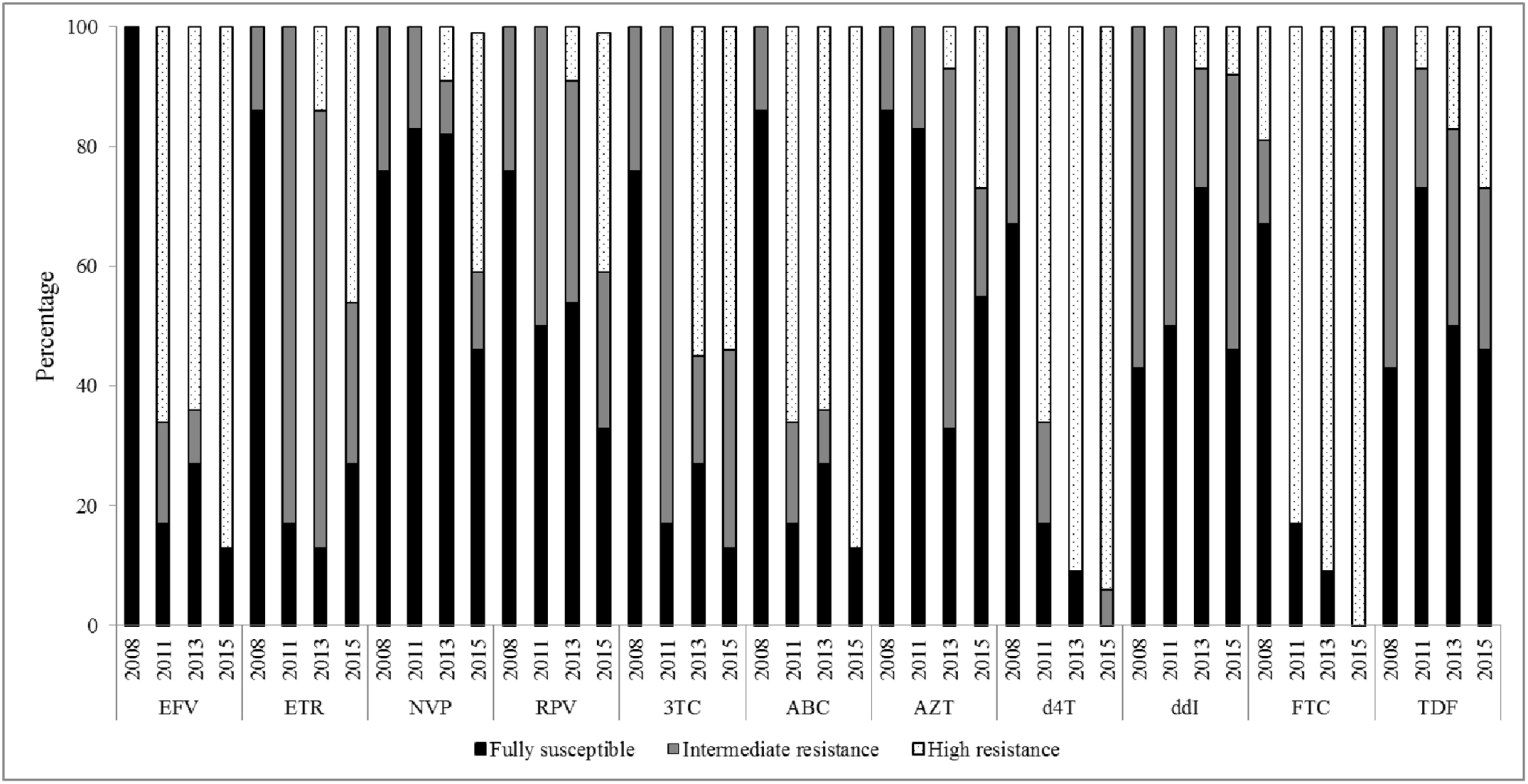
Predicted genotypic drug resistance susceptibility score over time. Keys: NNRTIs-efavirenz (EFV), etravirine (ETR), nevirapine (NVP), rilpivirine (RPV); NRTIs-lamuvidine (3TC), abacavir (ABC), zidovudine (AZT), stavudine (D4T), didanosine (ddl), emtricitabine (FTC), tenofovir (TDF).

At baseline, most of the viral isolates were fully susceptible for the available NRTIs and NNRTIs. However, the mean GSS for both all NRTIs and NNRTIs decreased overtime and the number of treatment option decreased overtime with viremia. Fig 5 shows the predicted genotypic drug susceptibility score of the available drugs in the clinical settings. For example susceptibility score of the common NNRTIs EFV has significantly reduced from 100% at baseline to 15% by the time point T3. On the other hand, among 100 consecutive HIV-1 infected adults who have been receiving ART since 2005 (Grroup-1), virological suppression at time point-T0 was observed in 82% of the patients on a median time of 24 months on ART [3]. Among the virological failed patients only one patient had K103N mutation which confers resistance to NNRTIs [3]. By a median time of 5 years on ART (in 2011), 76% of the patients were on ART of which 93% (71/76) were virologically suppressed. Again, by the median time of 8 years (in 2013) and 10 years (in 2015) years on ART, 94% (51/54) and 93% (29/31) of the patients, respectively were virologically suppressed. That means, the incidence of virological failure was almost stable overtime. Moreover, those who did not reach to virological suppression level had a low level viremia ranging from 1.7 to 2.5log_10_ copies /ml.

## Discussion

This study describes a decade virological outcomes and measures the incidence of virological failure and acquired drug resistance mutations in Ethiopian HIV-1C infected adults at 4 time points from the Horn of Africa. The study has the following main findings in relation to the incidence of virological failure, accumulation of acquired drug resistance mutations and the pattern of mutational pathways.

First, a relative upward trend in the incidence of both virological failure and acquired drug resistance mutation driven by NRTIs and NNRTIs is observed over the years supporting the notion that prolonged exposure to ART drives the emergence of mutant variants [5, 16] and are consistent with studies from other African countries where a significant increase in rate of both virological failure and drug resistance overtime since rollout of ART has been observed [9, 12, 16–21] and initial reports from resource reach countries [22]. Considering recent findings from the country that shows nearly 50% of patients on ART continue to engage in risky sexual practice [23–25], the increasing rate of virological failure with increased level of viremia may have an impact not only on increasing overall risk of HIV transmission at a population level but also on increasing rate of reinfection by resistant strains. Thus, both population-level and patients targeted interventions to reduce risky sexual behavior is warranted.

The higher rate of NRTI resistance may reflect the expected pattern of mutations with increasing use of first line therapy [26]. Moreover, the increasing accumulation rate of acquired drug resistance mutations could be due to the fact that almost all of the patients with virologic failure and drug resistance mutations had not met the WHO clinical and/or immunological failure criteria and continued the failing regimen and are consistent with recent findings which show a 30% per year increase in drug resistance in east Africa [19]. However, there is a significant difference in rate of virological failure and accumulation of drug resistance mutation among those who initiated ART at roll out (the first group of patients receiving cART in Ethiopia) and scale up of ART. This heterogenecity among these group of patients could be associated with task-shifting approach from more specialized (roll out of ART) to less specialized (scaling-up of ART) health workers although our study and previous studies showed that the latter is non-inferior to the former [13, 27, 28] in terms of care during the clinical visits and retention in ART care. The influence of task-shifting approaches on virological outcomes is not well examined and could result variation monitoring of ART and possibly influence patient’s adherence although overall adherence in this particular study and other several studies from sub-Saharan Africa is not worse compared to western countries [13, 28]. Thus, other drug related factors such as drug supply and regimens used [8] and local programmatic factors such as when to start/switch, what to start/switch, lack of access for viral load and resistance testing, lack of alternative drugs with high genetic barriers could have also contributed to the observed difference.

Second, the absence of major protease inhibitors drug resistant mutations in this cohort could explain the relation between drug access and emergence of drug resistant mutations. This could reflect the presence of limited access of the drug class in the country as less than 2% of patients are taking drug regimens containing this drug class. The occurrence of naturally occurring polymorphic changes on PR region may have clinical relevance as these changes may result in the evolution of drug resistance along distinct mutational pathways, or in the incidence of different pathways when considering long-term treatment strategies of HIV-1C infected patients with PI based regimen [29]. A rapid increase in lamivudine and TAMs were noted and could be associated with the extensive use of thymidine analogues and their eventual accumulation overtime in Africa as patients continue failing regimen which might reduce the effectiveness of second line TDF containing combinations. These findings are important in view of the fact that following WHO recommendations TDF is replacing thymidine analogues, phasing out of the nevirapine from first line regimens and considering LPV/r-based regimen as the PI of choice in second-line treatment in Africa. In this cohort empirical switching to second line therapy in small number of patients with failing regimen and existence of drug resistance mutations led to effective viral suppression demonstrating that TDF containing and PI-based second line regimens can successfully re-suppress NRTI and NNRTI resistant HIV mutants and improves virological outcomes. Thus, to preserve this recent WHO recommendation and to protect resistance development against TDF and PIs, pre-ART resistance testing for key NNRTI and PI mutations could be considered. Third, the failure of the WHO public health clinical and immunological approaches in detecting treatment failure support the recent WHO recommendation for HIV-1 viral load testing in Africa [15] but yet less than 2% of patients had VL tested [22] and still in some countries like Ethiopia non-existing in routine clinical practice.

Lastly, the study in one side identified an increasing common mutational pathways overtime with two thymidine non-analog (M184V and K65R) and three thymidine analog (D67N, K70R and K219E) major nucleoside reverse transcriptase inhibitor (NRTI)-associated DRMs and four major NNRTI-associated DRMs (K103N, Y181C, G190A, and V106M) which is consistent with previous studies [20, 29]. But on the other side, a stable rate of selection was also observed for some NRTI mutations (such as Y115F, L210W) and several NNRTI mutations (such as L100I, K101E, P225H and M230L). Thus, the first common mutational pathways could be important for designing a point of care genotypic resistance test as suggested before [30] and would be useful in the region.

## Conclusions

In summary, an upward trend of both virological failure and NRTI and NNRTI associated drug resistance acquired drug resistance mutations has been observed overtime. The findings indicate that ART programs in east Africa could not achieved WHO suggested targets of long term virological suppression overtime. Thus, regional focused strategies to decrease the rate of virological failure and acquired drug resistance, to prevent the spread of mutant variants, to increase access for TDF, PI drugs, HIV viral load and resistance testing are needed if the 90-90-90 targets are to be attained. As a result treatment success of current first-line ART could be improved, the efficacy of second-and third-line therapies could be preserved and transmission of resistant variants which could be the future challenge of the region unless controlled early could be prevented.

## Supporting information

S1 Table Clinical characteristics & baseline transmitted drug resistance (at time point-T0): Reprinted from {Mulu 2014 #57} Mulu *et al*. *BMC Infectious Diseases* 2014 14:158 10.1186/1471-2334-14-158. (DOC)

S2 Table Clinical characteristics and acquired antiretroviral drug resistance mutations (at time point-T1): Reprinted from {Mulu 2015 #53} Mulu *et al*. PLoS One. 2015 Oct 29;10(10):e0141318. 10.1371/journal.pone.0141318. (DOC)
