# Supplementary material for: Upward trends of acquired drug resistances in Ethiopian HIV-1C isolates: A decade longitudinal study
Source: PLoS One. 2017 Oct 19;12(10):e0186619. doi: 10.1371/journal.pone.0186619 (PMC5648217; doi:10.1371/journal.pone.0186619)
Supplement: S1 Table — (DOC) [file pone.0186619.s001.doc]

**S1 Table**: Clinical characteristics &baseline transmitted drug resistance (**at time point-T0**)

| NRTI NNRTI  Age/Sex HIV load* ------------------------------------------------ --------------------------------------------  *Mutation Resistance to Mutation Resistance to* |
| --- |
| 25 /F 5.44 - - *E138G* ETR |
| 35/F 4.20 - - **Y188H** ETR,DLV,EFV,NVP |
| 35/F 4.58 **K219E** AZT,D4T,ABC,DDI,TDF - - |
| 30/F 3.88 **L210W** AZT,D4T - - |
| 28/F 5.76 - - *E138G* ETR |
| 35/F 5.49 - - *E138A* ETR |
| 30/F 4.31 **K65R** ABC,ddI,FTC,3TC,d4T,TDF - - |
| 40/F 5.50 - - *E138A* ETR |
| 45/F 4.91 - - *E138A* ETR |
| 27/F 4.49 - - *E138A* ETR |
| 27/F 4.38 - - *V90I* ETR |
| 44/F 4.64 - - *V90I* ETR |
| 25/M 5.94 - - **K101E**, *E138A* DLV,EFV,ETR,NVP |
| 32/M 5.17 - - **G190A** EFV,ETR,NVP,RPV |
| 30/M 5.49 - - *E138A* ETR |
| 22/M 5.13 - - *E138A* ETR |
| 35/M 4.80 - - *E138A* ETR |
| 28/M 4.82 - - **G190A** EFV,ETR,NVP,RPV |
| 38/M 5.17 **L210W** AZT,D4T - - |
| 22/M 5.12 - - V90I ETR |
| 24M 4.36 - - **M230I** RPV |

*HIV load in log_10_ copies/ml; Mutations in bold are only considered by both IAS and Stanford University HIV drug resistance algorithm; Mutation in *Italics* are reported by IAS only. *Abbreviations*: *3TC* lamiduvine, *ddI* didanosine, *d4T* stavudine, *FTC* emtricitabine, *TDF* tenofovir, *ZDV* zidovudine, *NNRTI* non-nucleoside RT inhibitors, *DLV* delavirdine, *EFV* efavirenz, *ETR* etravirine, *NVP* nevirapine, *RPV* rilpivirine.

Reprinted from {Mulu 2014 #57} Mulu *et al.BMC Infectious Diseases* 2014 **14**:158 doi: 10.1186/1471-2334-14-158
