# Supplementary material for: Upward trends of acquired drug resistances in Ethiopian HIV-1C isolates: A decade longitudinal study
Source: PLoS One. 2017 Oct 19;12(10):e0186619. doi: 10.1371/journal.pone.0186619 (PMC5648217; doi:10.1371/journal.pone.0186619)
Supplement: S2 Table — (DOC) [file pone.0186619.s002.doc]

**S2 Table: Clinical characteristics and acquired antiretroviral drug resistance mutations (at time point-T1)**

| Age/  Gender | CD4^+^ T cells | | HIV RNA | | Baseline ART | Time on ART | NRTI | | NNRTI | |
| --- | --- | --- | --- | --- | --- | --- | --- | --- | --- | --- |
|  | T0 | T1 | T0 | T1 |  |  | mutation | resistant | mutation | resistant |
| 51/M | 250 | 391 | 26,915 | 19,952 | 3TC+D4T+EFV | 25 | M184V | 3TC, FTC | K103N | EFV, NVP |
| 28/M | 401 | 449 | 14,791 | 2,511 | 3TC+D4T+EFV | 35 | M184V | 3TC, FTC | K103N | EFV, NVP |
|  |  |  |  |  |  |  |  |  | V106M | EFV, NVP, ETR |
| 25/F | 229 | 393 | 26, 9153 | 91,201 | 3TC+D4T+NVP | 29 | None | - | Y181SY | NVP |
| 38/M | 8 | 276 | 9,120 | 91,201 | 3TC+D4T+NVP | 33 | K65R | 3TC,DDI,FTC, TDF | Y188L | EFV, NVP |
| 25/F | 32 | 270 | 158 | 4,786 | 3TC+D4T+NVP | 29 | M184V | 3TC, FTC | V90I | EFV, NVP, ETR |
|  |  |  |  |  |  |  |  |  | K101E | EFV, NVP, ETR |
|  |  |  |  |  |  |  |  |  | G190A | EFV, NVP, ETR |
| 29/F | 199 | 281 | 26,302 | 53,703 | 3TC+D4T+EFV | 35 | M184V | 3TC, FTC | None | - |
| 38/F | 274 | 319 | 1,071 | 26,915 | 3TC+D4T +NVP | 29 | None | - | None | - |
| 30/M | 32 | 299 | 5,754 | 97,923 | 3TC+D4T+NVP | 31 | None | - | None | - |
| 42/F | 75 | 474 | 33,414 | 442 | 3TC+D4T+NVP | 28 | None | - | None | - |
| 40/M | 191 | 426 | 80,013 | 553 | 3TC+D4T+NVP | 32 | None | - | None | - |
| 38/F | 157 | 387 | 51,833 | 889 | 3TC+D4T+NVP | 36 | None | - | None | - |
| 50/F | 95 | 416 | 103,443 | 1,570 | 3TC+D4T+EFV | 34 | None | - | None | - |
| 40/F | 170 | 383 | 19,059 | 1,886 | 3TC+D4T+EFV | 32 | None | - | None | - |
| 51/M | 112 | 463 | 11,433 | 871 | 3TC+D4T+NVP | 30 | None | - | None | - |
| 35/M | 211 | 301 | 5,635 | 1,471 | 3TC+D4T+EFV | 29 | None | - | None | - |

Age: in years; F: Female, M: Male; CD4^+^ T in cells/mm^3^; HIV RNA in copies/ml; Time on ART in months; NRTI (Nucleoside RT inhibitors): 3TC (lamiduvine), ddI (didanosine), d4T (stavudine), FTC (emtricitabine), TDF (tenofovir), ZDV (zidovudine); NNRTI (non-nucleoside RT inhibitors): EFV (efavirenz), ETR (etravirine), NVP (nevirapine); Amino acids: A (alanine), E (glutmatate), G (glucine), K (lysine), L (leucine), M (methionine), N (asparganine), S (serine), V (valine), Y (tyrosine)

Reprinted from {Mulu 2015 #53} Mulu *et al.*PLoS One. 2015 Oct 29;10(10):e0141318. doi: 10.1371/journal.pone.0141318
